# Glenoid Augmentation With Humeral Head Autograft in Reverse Shoulder Arthroplasty: A Retrospective Study of an Innovative Technique

**DOI:** 10.7759/cureus.100706

**Published:** 2026-01-03

**Authors:** Hayley Pennock, Avinash Rai, Richard Jeavons, Raymond Liow, Rachael Hine, Emma Tindall

**Affiliations:** 1 Trauma and Orthopaedics, Queen Elizabeth Hospital, Gateshead, GBR; 2 Trauma and Orthopaedics, NHS Greater Glasgow and Clyde Hospital, Glasgow, GBR; 3 Trauma and Orthopaedics, University Hospital of North Tees, Stockton-on-Tees, GBR; 4 Orthopaedics, University Hospital of North Tees, Stockton-on-Tees, GBR

**Keywords:** bone graft, glenoid augmentation, glenoid bone defect, glenoid bone loss, humeral head autograft, reverse shoulder arthoplasty

## Abstract

Introduction

The use of reverse shoulder arthroplasty (RSA) is expanding for both primary and revision procedures. Advances in implant technology and preoperative planning have improved the ability to manage complex glenoid defects. Various options are available for addressing severe glenoid bone loss, including eccentric reaming, glenoid reconstruction with bone graft, and the use of augmented glenoid baseplates. However, metal augments are often expensive and lead to more waste and CO₂ emissions, adding to our carbon footprint. Humeral head autografts are an excellent alternative, as they preserve bone and incorporate very well. We conducted a retrospective study and described a new innovative technique when using humeral head autograft for glenoid bone loss in reverse shoulder arthroplasty.

Methods

A retrospective review of 24 patients who underwent glenoid augmentation using humeral head autografts for reverse shoulder arthroplasty from 2017 to 2024. The majority of the patients were female, with a mean age of 75.4 years. All patients had a preoperative CT scan to assess glenoid bone loss and were classified as per Walch's classification. All patients were operated on by two consultant shoulder surgeons. A femoral head allograft was available for all as a standby in case of poor bone quality in the humeral head; however, this was only used in one patient. The deltoid-splitting McKenzie approach and a Delta Xtend reverse shoulder system (DePuy) were used in all patients. The humeral head was prepared to match the glenoid defect. The graft was held in place with a K-wire, followed by Metagelene; a log peg was used in most cases. Clinical and radiological outcomes were assessed retrospectively.

Results

Most patients (15) had central glenoid wear A2 as per the Walch classification. There was only one intraoperative complication of a calcar fracture, with no immediate postoperative complications. The average duration of follow-up was 48.8 months. All patients had significantly better range of motion postoperatively and an improvement in Oxford Shoulder Score. A total of 22 patients did not have any pain in the shoulder at the last clinic follow-up, with 100% patient satisfaction following the surgery. Graft was incorporated in all but two patients (89.5%). No patients required revision surgery.

Conclusion

Our new technique described for glenoid augmentation with humeral head bone graft is an effective technique for addressing glenoid bone defects in reverse shoulder arthroplasty, with good functional outcomes at long-term follow-up.

## Introduction

The use of reverse shoulder arthroplasty (RSA) is expanding for both primary and revision procedures. The indications include cuff tear arthropathy, proximal humeral fractures, irreparable rotator cuff tears, inflammatory arthropathy, and glenohumeral joint osteoarthritis [1]. If not carefully assessed and appropriately managed, glenoid bone loss can present significant challenges and result in several complications. It is reported that 40% of patients undergoing shoulder arthroplasty have acquired glenoid bone loss and deformity [2,3]. Studies have established that preoperative posterior glenoid erosion is not only a risk factor for glenoid loosening but also results in worse outcomes for function and pain [4]. 

Inability to recognise the severity and amount of glenoid bone loss preoperatively can result in incorrect positioning and fixation of the glenoid component, which in turn can increase stress forces across the implant, resulting in implant micromotion and loosening [5]. It can also lead to dislocation and instability, poor functional outcomes, and decreased bone stock for future revision procedures [6]. 

To address this issue, different surgical techniques can be implemented, including eccentric reaming, the use of augmented glenoid baseplates, and glenoid reconstruction with bone graft [7]. A systematic review by Lanham et al. [8] showed that glenoid bone grafting and augmented baseplates have similar overall clinical outcomes, complication rates, and revision rates. However, metal augments are often expensive and lead to more waste and CO₂ emissions, adding to our carbon footprint [9].

Our focus for this study is to describe a new innovative technique that is reproducible for glenoid augmentation using humeral head autograft in primary RSA. The primary objective of this study is to evaluate the clinical and radiological outcomes, focusing on graft incorporation, complications, and functional parameters at medium- to long-term follow-up.

Consequences of glenoid bone loss 

Causes of glenoid bone loss in the native shoulder include rotator cuff tear arthropathy, failed rotator cuff repairs with superior humeral head migration, osteoarthritis, osteoarthritis with rotator cuff failure, fracture sequelae, or chronic glenohumeral dislocation [10]. 

The surface of a standard glenoid baseplate that interfaces with the glenoid bone is oriented perpendicular to the central axis of the main fixation screw or peg. In the case of severe glenoid bone loss, a flat baseplate has an extremely low sitting percentage and does not provide adequate stability [11]. Consequently, in cases where the glenoid exhibits substantial bone loss or deformity, the available bony surface may not provide optimal contact for proper implantation of a standard baseplate [12]. If the alignment between the glenoid surface and the central screw is not perpendicular, portions of the baseplate’s backside may fail to contact the native bone. This lack of contact can pose a considerable surgical challenge, as it compromises secure baseplate fixation [13]. Approaches to manage glenoid bone loss include eccentric reaming to remove excess bone on the elevated side for a flush implant fit, augmented or lateralised baseplate, or bone grafting to restore stock. 

If fixation is suboptimal, this can result in increased micromotion. Bone ingrowth is unlikely beyond 150 micrometres of motion and will lead to gross loosening and eventually failure of the implant [14]. Implant micromotion can contribute not only to loosening but also to rotational instability, which may result in implant dislocation or component dissociation [6]. 

Bone grafting is particularly useful for correcting large defects, offering a cost-effective and adaptable solution that can address deficiencies across multiple regions [15]. 

Glenoid bone loss is associated with higher rates of complications and revisions due to factors such as inadequate soft-tissue tensioning, glenoid instability, and malalignment, which can contribute to early loosening, component impingement, and failure, often compounded by diminished bone stock for future revisions [16]. Some studies report nearly a tenfold increase in failure rates with glenoid bone loss as compared to patients without such bone loss [17,18]. 

Moreover, there is a decreased deltoid wrapping effect in the presence of glenoid bone loss, further reducing the stability of the prosthesis [19]. 

Techniques of addressing glenoid bone loss 

After identifying glenoid bone loss, several management strategies can be employed, including eccentric reaming, orienting the baseplate along an alternative scapular centreline, bone grafting, or using metallic augmentation with either standard or custom baseplates. For more complex reconstructions, patient-specific instrumentation and navigation may also be used [20]. 

Early methods described to manage eccentric bone loss include eccentric reaming. However, in severe deformities, substantial amounts of bone can be removed from the subchondral plate, which can be detrimental. Further disadvantages include joint line medialisation and removal of too much bone stock, which can complicate future revision surgery [21]. In cases with mild-to-moderate bone loss and retroversion or anteversion, eccentric reaming remains a useful option. This is a simple technique which is cost- and time-effective [20]. Recent studies are limited in terms of high-quality evidence, with hybrid techniques being described, such as the use of eccentric reaming with bone grafting. In cases with more substantial bone loss, other reconstructive options should be considered [7].

Traditionally, the glenoid baseplate is positioned along the bisector of the glenoid vault, also known as the anatomic centre line [22]. In cases of severe glenoid bone loss, the remaining bone along this line is often insufficient to achieve secure primary fixation of the baseplate’s central screw or post [20]. Klein et al. introduced orienting the baseplate with selective anteversion and inferior tilt, allowing the central fixation feature to engage the bone at the base of the scapular spine [2]. This approach may be combined with limited corrective reaming. While the ideal proportion of baseplate supported by bone is not precisely defined, most surgeons aim for at least 50-80% support, depending on bone quality and fixation type (post vs. screw) [5]. This technique favours adequate baseplate fixation over anatomic component positioning [2,23], which could lead to malposition and imbalance, leading to complications.

In cases of severe glenoid bone loss, deformity correction may not be achievable with asymmetric reaming and standard implants due to excessive medialisation and poor fixation in weak cancellous bone. In such situations, posterior bone grafting or metallic augments are commonly used as effective strategies for managing glenoid bone loss and deformity, offering improved clinical outcomes with comparatively low rates of complications and revisions [8,12]. Bone grafting is low-cost and has the flexibility to correct large and multiple defects [15]. 

Both autografts and allografts can be used; however, humeral head autografts are usually preferred due to their immediate availability, lack of cost, and lack of donor site morbidity [24]. Allografts, which may be fresh-frozen or freeze-dried and sourced from sites such as the femoral head, humeral head, shaft, or tibia, typically need to be pre-ordered from a bone bank and thawed before surgery [25]. 

Boileau et al. described the Bony Increased Offset for Reversed Shoulder Arthroplasty (BIO-RSA) technique, where the bone graft is placed underneath the whole baseplate, lateralising the centre of rotation, allowing for biplanar angular correction [26]. Restoration of joint alignment, muscular tension, and stability can be achieved while enhancing mechanical fixation, with the added benefit of lateralisation that can improve the impingement-free range of motion of the RSA construct [27]. Results up to 10 years postoperatively have shown to reliably correct multiplanar glenoid deformity > 25°, with good functional outcomes and low revision rates [28]. 

Another viable alternative includes the use of a femoral head allograft. Castricini et al. (2024) evaluated 20 RSAs with severe glenoid deformities, reporting a median preoperative version of 21.8° in retroverted and -13.5° in anteverted glenoids, and demonstrated restoration of shoulder function, 85% graft incorporation, and no revisions at a median follow-up of 26.5 months [29]. However, problems can arise with graft incorporation in allografts; Paul et al. [30], in their review, demonstrated a higher rate of union with autologous grafting (97% for autograft bone versus 90% for allograft bone). 

Glenoid bone grafting offers several advantages, including cost-effectiveness, preservation and restoration of the native glenoid bone stock, and valuable bone stock for any future reconstructive procedures in the event of implant failure. However, it may not be reproducible due to variability in graft contouring and can be technically challenging [5,31]. Other disadvantages include failure of graft incorporation, resorption, non-union, baseplate fixation failure, and increased operative time [32,33]. 

Indications for the use of augmented baseplates are in keeping with those for the use of bone grafting. Augmented baseplates are produced in various sizes and shapes (flat, full wedge, half wedge) that allow placement in various locations to maximise bony contact and fixation. Since the size/shape of the augment is predetermined, they may not fit the defect perfectly and not allow complete correction of the deformity. Proper orientation is crucial, as only a few degrees of malrotation can significantly reduce contact with native bone [34]. Reduced contact increases the risk of micromotion, aseptic loosening, and the need for future revision surgery [18].

These challenges may be overcome with the use of custom-specific 3D-printed baseplates [20]. Custom shoulder-specific metallic glenoid baseplates are created using preoperative CT imaging and are tailored to the surgeon’s specifications in collaboration with the manufacturer’s engineering team [35]. 

The advancement of custom-made orthopaedic implants offers promising solutions for managing severe glenoid bone loss. Progress in 3D printing technology has led to the manufacture of an implant that matches, as accurately as possible, the glenoid deformity [36]. Patient-specific instrumentation (PSI) improves the accuracy of component implantation as planned in the preoperative analysis. Although early data appear promising, long-term data on augmented components are limited [20]. Furthermore, these options are more expensive than bone grafts.

## Materials and methods

This was a retrospective review of the humeral head autograft performed for treatment of posterior glenoid bone loss with primary reverse shoulder replacement in a single institution, the University Hospital of North Tees, by two surgeons from 2017 to 2024. Inclusion criteria for this study included primary glenohumeral osteoarthritis with posterior glenoid bone loss, complete preoperative and postoperative datasets, and imaging studies. 

All patients had a preoperative CT scan to assess glenoid bone loss and were classified as per the modified Walch classification [37]. The Modified Walch classification describes glenoid morphology based on CT imaging; categories include A1 and A2, which describe concentric wear; B1, B2, and B3, which describe eccentric wear; C, which describes glenoid retroversion of at least 15 degrees; and D, which describes glenoid anteversion or anterior humeral head subluxation. 

All preoperative, intraoperative, and postoperative records and imaging were reviewed. Patient demographics, complications, and clinical and radiographic findings were identified. The primary outcome measure was the Oxford Shoulder Score (OSS). Formal permission was granted to use the OSS in this study from Oxford University Innovation. The OSS is a validated patient-reported outcome measure used to evaluate shoulder pain and functional ability [38,39]. The secondary outcome measures were range of motion, complications, and graft incorporation. 

All patients were operated on by two consultant shoulder surgeons. A femoral head allograft was available for all as a standby in case of poor bone quality in the humeral head; however, this was only used in one patient. 

Surgical technique 

**Figure 1:**
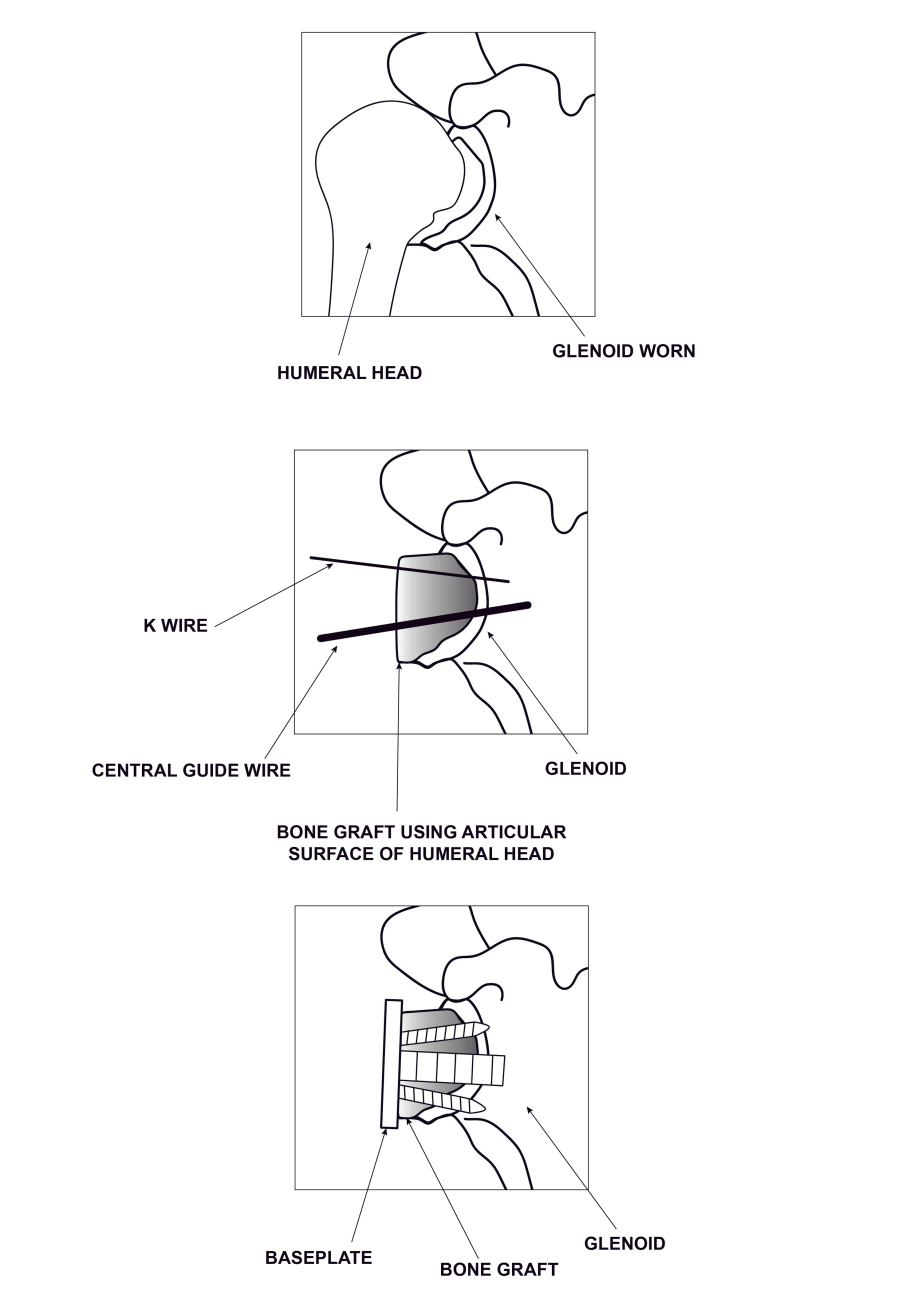
Illustration of bone graft technique Original image designed by 'Rebecca Pennock Design' (Individual freelance graphic designer). Permission granted to use image for this article (relationship: sister to the first author).

The deltoid-splitting McKenzie approach was used in all patients. The long head of the bicep’s tendon was identified and tenodesed to the superior border of the pectoralis major insertion. The Delta Xtend reverse shoulder system (DePuy) was used in all patients. Care was taken to protect the axillary nerve at all times. The humeral head was resected using jigs as per standard technique. The glenoid was prepared and reamed, and the defect was assessed to prepare the graft from the humeral head. We hypothesised that the humeral head is responsible for the defect and would be the best match to fit in the defect (Figure 1). Flipping the graft compared with the standard technique allows the humeral head to fit more congruently into the defect, decreasing shear forces and edge loading, and thereby lowering the risk of graft fragmentation.

**Figure 2:**
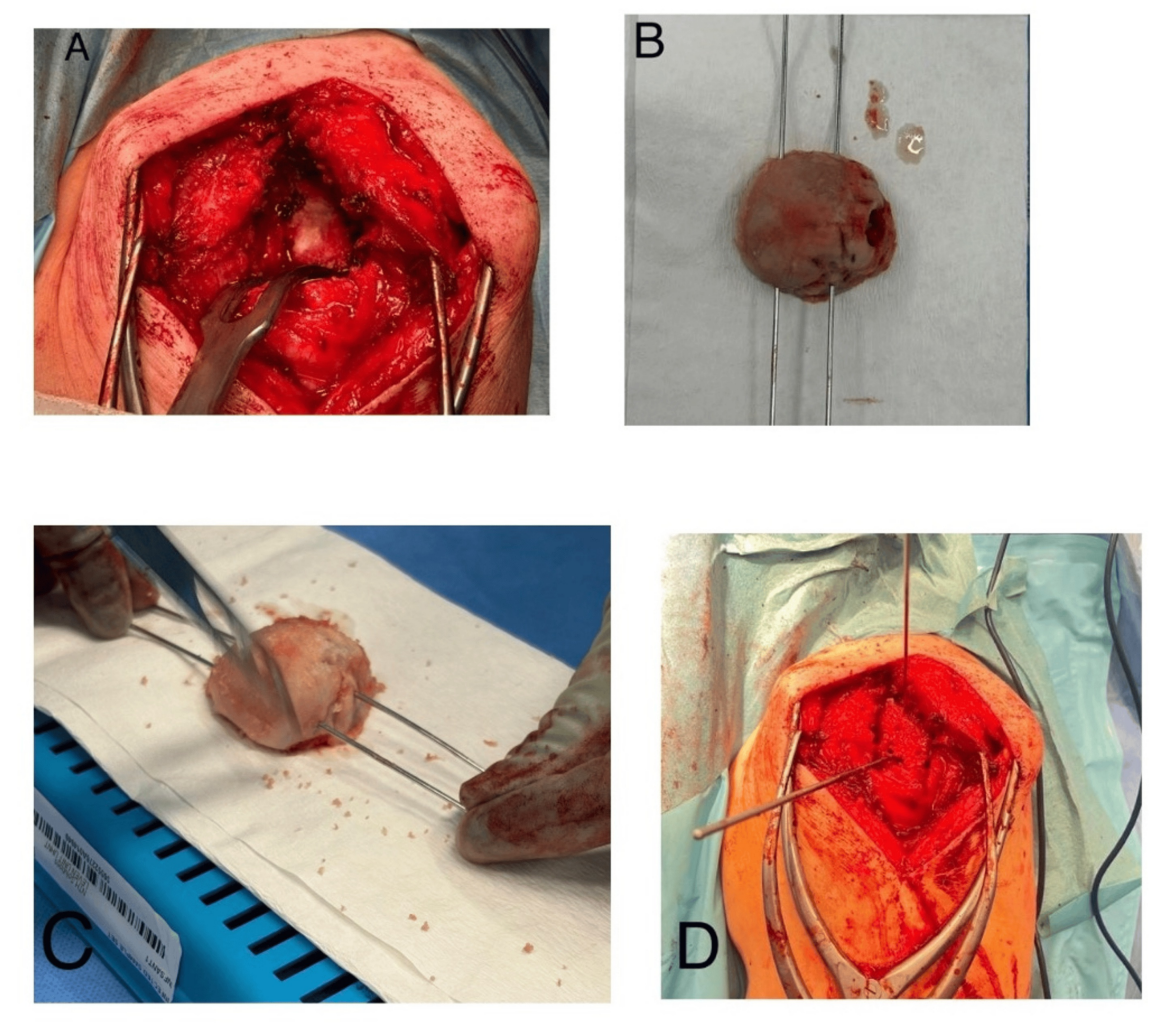
Intraoperative surgical steps demonstrating surgical technique A: Preparation of glenoid, B: Humeral head graft prepared using parallel wires to match the defect; C: Cartilage removed from humeral head using saw; D: Humeral head secured using superior K-wire with central guide in place for the preparation of metaglene

An oscillating saw and nibblers were used to remove the articular cartilage. Once prepared, the graft was used to fill the defect and held using K-wires placed superiorly (Figure 2).

Another central guide wire was placed using a jig for the baseplate, followed by reaming for the central peg. A long peg was preferred to ensure a good hold of the metaglene. A standard peg is 13.5 mm in this system, and longer pegs are available in 23.5 or 28.5 mm sizes. The graft allowed filling of the defect to restore the offset, and the central guide wire position also allowed for inferior tilt of the baseplate, thereby reducing the risk of scapular notching. Following this, the glenosphere was trialled and the humerus prepared using the standard procedure. 

Statistical analysis was done using Microsoft Excel (Redmond, USA). Continuous variables (e.g., OSS) were summarised with means ± standard deviation for normally distributed variables or medians for non-normal distributions. Categorical variables (e.g., gender, classification type) were reported as percentages. Paired Student t-tests were used to analyse associations between continuous variables.

## Results

A total of 24 patients underwent glenoid augmentation using humeral head autografts for RSA from 2017 to 2024 using this technique. The majority (75%) of the patients were females (18 females, six males), and the mean age of the cohort was 75.4 years (52.4-89.3 years). 

Most patients (62.5%) had central glenoid wear A2 as per the modified Walch classification (Table 1).

**Table 1:** Distribution as per Modified Walch classification

Type	Percentage
A1	4.2% (1)
A2	62.5% (15)
B2	12.5% (3)
B3	12.5% (3)
D	8.3% (2)

The average duration of follow-up was 48.8 months (9.5-92.1 months). Four patients were followed up for at least five and a half years (67.8-92.1 months). 

All patients had significantly better range of motion postoperatively and an improvement in OSS (Table 2).

**Table 2:** Clinical and functional outcomes SD: standard deviation

	Preoperative	Postoperative	p-value (t test)
Forward flexion (degrees)	58.3 (SD-25.1)	110.2 (SD-32.3)	<0.001
Abduction (degrees)	51.7 (SD-20.9)	100.6 (SD-26.4)	<0.001
Oxford shoulder score	17.7 (SD-3.9)	36.8 (SD-10.3)	<0.001

A total of 22 patients did not have any pain in the shoulder at the last clinic follow-up. Graft incorporation was assessed using standard radiographs - Grashey view, scapular Y view, and modified axillary view. Graft was incorporated in all but two patients (89.5%). One patient with failed graft incorporation had a fall in the immediate postoperative period, which may have contributed to this, but reported no clinical concerns when followed up. None of the patients required revision surgery. They remain under regular follow-up. 

There was one intraoperative complication of a calcar fracture in the humerus, with no immediate postoperative complications. 

## Discussion

Glenoid bone loss is a major problem that needs to be recognised preoperatively. There are multiple options which could be used to address glenoid bone loss; however, bone grafting shows good results and is cost-effective and sustainable, making it a superior choice. 

In cases of mild bone loss, glenoid bone grafting can still be used to augment the glenoid bone stock, correct the RSA angle, and avoid further bone loss in potential future revision surgery [20]. Boileau et al. described the bony increased offset (BIO) technique, in which the implant compresses the bone graft to promote graft incorporation and achieve lateralisation of the glenohumeral centre of rotation [27]. 

Guareschi et al. demonstrated that patients undergoing primary RSA with humeral head autograft for severe glenoid bone loss experienced improvements in postoperative range of motion and functional outcome scores that were significant but inferior to those of matched controls. Suggesting that reconstruction with humeral head autograft is a reasonable and effective strategy for patients with significant glenoid bone loss [17]. 

Bone grafting with both autografts and allografts has been shown to be effective in patients undergoing RSA with large glenoid defects. Paul et al. [30] conducted a systematic review of 11 studies and found 95% evidence of radiological union (97% when using autograft bone), consistent improvement in range of motion and functional outcomes, with a 2% revision rate and an 18% complication rate. This is comparable to our results, which echo the same findings using our technique. 

Contraindications to humeral autograft have been described by Ingoe et al. [40] and include absolute contraindications such as tumour and infection, and relative contraindications such as osteopenia and avascular necrosis. None of our patients had these contraindications. 

Trauma and orthopaedics is recognised as a major contributor to the hospital carbon footprint, with the NHS producing four to five per cent of the nation’s total greenhouse gas emissions. The NHS produces a quarter of all public sector waste, with operating rooms generating almost a third of a hospital's total waste. 40% of operating room waste is from packaging materials. The current focus is on sustainability and the reduction of waste, in particular implant waste [9,41]. We hypothesise that utilising humeral head bone grafts and not using glenoid augmented prosthesis results in a reduction of waste production and carbon emissions from manufacturing and transport of the prosthesis.

Standard and angled BIO-RSA techniques have been described to address glenoid bone loss. Bone grafts used for the glenoid baseplate construct can be shaped freehand or using instrumentation such as the Stryker Tornier BIO-RSA system [42]. Harmsen et al. [43] found 100% graft incorporation with ‘shaped’ humeral head autografts in patients undergoing RSA. Their technique describes a variation of the BIO-RSA technique with shaping of the humeral head autograft using a microsagittal saw. At an average follow-up of 34.6 months, there was improvement in mean functional outcome scores, active range of motion, and strength. Out of 29 patients, there were two complications due to infection, with one requiring revision for deep infection. Limitations include the small sample size and single surgeon operating, including shaping of the harvested bone graft using a micro-sagittal saw, which may be difficult to consistently reproduce with the same outcome. 

Ingoe et al. [40] described the technique of on-the-table shaping of a femoral allograft to match the glenoid defect using a handheld saw and high-speed burr. Graft healing was 92%, with two cases of graft resorption with glenoid component loosening at one and two years. They also reported one periprosthetic humeral fracture, one case of instability, one acromial spine fracture, and one case of persistent ulnar nerve symptoms. Limitations to this study again include a small sample size and a follow-up of two years only.

Jones et al. [12] further described a technique of shaping allografts and autografts by hand to match the glenoid defect. They then used custom inverse reamers to ream the back of the graft to match the reamed native glenoid surface more accurately. Allograft bone matrix gel was used to fill any small gaps. The graft was held in place using K-wires and then further reamed to accept the baseplate. They reported excellent postoperative outcomes in both allograft and autograft groups; however, these results did not equal the results of the cohort undergoing RSA without the need for bone grafting. Their complication rate was 13.6%, with two graft failures requiring revision, two infections, and one postoperative dislocation. Limitations include the fact that only standard radiographs were used to assess graft incorporation as opposed to advanced CT imaging. In addition, as compared to our technique, their technique may be difficult to replicate and requires the use of allograft bone matrix as well. 

Tashjian et al. [16] again used a similar technique whereby an oscillating saw was used to carve the humeral head to match the glenoid defect; this was then held in place with K-wires, and the glenoid was prepared accordingly as per the manufacturer's instructions, depending on which shoulder system was used. One significant limitation reported was the ability to accurately measure the defect size, and again, it was relatively difficult to match the defect as compared to our technique. 

Our study describes a new technique for the utilisation of the humeral head autograft to match the anatomy of the patient, allowing it to be more easily reproducible when compared to previous studies, which tend to involve freehand shaping of the graft using a handheld saw. Using our technique, we had no immediate postoperative complications, with two patients developing loosening around the glenoid component on postoperative imaging. Despite this, these patients had no postoperative pain, and they were satisfied with their surgery. No patients required revision surgery. There was a significant improvement in postoperative range of motion, OSS, and 100% patient satisfaction at follow-up. 

Strengths of our study include the description of a new, detailed, reproducible surgical technique. Use of a single RSA system and consecutive cases with two consultant shoulder surgeons, which reduces technical variability. This study also benefits from the use of a validated scoring system (OSS) and long-term follow-up of patients. Limitations to our study include our small sample size, which reduces the reliability of our study and limits the generalisability of our findings to the larger population. The retrospective nature of the study results in the potential for bias, reliance on the accuracy of pre-existing data, and confounding from other unmeasured factors. Not using a control group also means that our conclusions are weaker and less valid than if a control group were used.

## Conclusions

Our new technique, described for glenoid augmentation with humeral head bone graft, is an effective technique for addressing glenoid bone defects in RSA, demonstrating good radiological outcomes and high rates of patient satisfaction, with excellent outcome scores achieved with more than five years of follow-up. This technique is an excellent option if bone grafting is not contraindicated.

We hypothesise that recycling the humeral head and reducing the usage of augmented baseplates and implants results in less waste production and a reduction in carbon emissions from the manufacturing and transport of prostheses. However, further research is needed to accurately assess this outcome.
